# Effect of the Fermented Soy Q-CAN^®^ Product on Biomarkers of Inflammation and Oxidation in Adults with Cardiovascular Risk, and Canonical Correlations between the Inflammation Biomarkers and Blood Lipids

**DOI:** 10.3390/nu15143195

**Published:** 2023-07-19

**Authors:** Sarah M. Jung, Amandeep Kaur, Rita I. Amen, Keiji Oda, Sujatha Rajaram, Joan Sabatè, Ella H. Haddad

**Affiliations:** 1 Center for Nutrition, Healthy Lifestyle and Disease Prevention, Loma Linda University, Loma Linda, CA 92350, USA; sjung15@calstatela.edu (S.M.J.); akaur1@llu.edu (A.K.); ramen@students.llu.edu (R.I.A.);; 2 Rongxiang Xu College of Health and Human Services, California State University Los Angeles, Los Angeles, CA 90032, USA

**Keywords:** fermented soy product, hsCRP, IL-1β, IL-1Ra, TNF-α, haptoglobin, total antioxidant status, TBARS, canonical correlation

## Abstract

Systemic low-grade inflammation plays a key role in the development of cardiovascular disease (CVD) but the process may be modulated by consuming fermented soy foods. Here, we aim to evaluate the effect of a fermented soy powder Q-CAN^®^ on inflammatory and oxidation biomarkers in subjects with cardiovascular risk. In a randomized crossover trial, 27 adults (mean age ± SD, 51.6 ± 13.5 y) with a mean BMI ± SD of 32.3 ± 7.3 kg/m^2^ consumed 25 g daily of the fermented soy powder or an isoenergic control powder of sprouted brown rice for 12 weeks each. Between-treatment results showed a 12% increase in interleukin-1 receptor agonist (IL-1Ra) in the treatment group, whereas within-treatment results showed 23% and 7% increases in interleukin-6 (IL-6) and total antioxidant status (TAS), respectively. The first canonical correlation coefficient (r = 0.72) between inflammation markers and blood lipids indicated a positive association between high-sensitivity C-reactive protein (hsCRP) and IL-1Ra with LDL-C and a negative association with HDL-C that explained 62% of the variability in the biomarkers. These outcomes suggest that blood lipids and inflammatory markers are highly correlated and that ingestion of the fermented soy powder Q-CAN^®^ may increase IL-1Ra, IL-6, and TAS in individuals with CVD risk factors.

## 1. Introduction

Systemic chronic inflammation is a contributing factor to cardiovascular disease (CVD) and metabolic disorders [1]. With the increasing prevalence of these conditions, identifying potential dietary factors linked to decreased risk of inflammation is an important research priority. Dietary factors such as fiber, polyphenols, and unsaturated lipids have been associated with lower concentrations of inflammatory markers, whereas other factors such as saturated fat have been associated with higher levels of inflammation [2].

A major oilseed crop, the soybean (*Glycine max*) is also a nutritious legume abundant in plant protein, unsaturated fat, and bioactive compounds [3]. Soy consumption has long been linked with lower rates of chronic diseases and various health benefits [4,5]. Multiple classes of bioactive compounds are found in soy with isoflavones (particularly genistein and daidzein) receiving attention with regard to inhibition of inflammation [6]. Soy isoflavones were first shown to exert anti-inflammatory properties and lower circulating levels of cytokines in in vivo and animal models [7,8,9]. In population studies, soy food consumption was related to lower circulating levels of tumor necrosis factor-alpha (TNF-α), interleukin-6 (IL-6), and soluble tumor necrosis factor (TNF) receptors in middle-aged Chinese women [10] and data from the National Health and Nutrition Examination Survey (NHANES) 2005–2008 showed an association between lower circulating C-reactive protein (CRP) concentrations and higher urine excretion of isoflavonoids [11].

Subsequently, multiple human clinical trials tested various types, schedules, and doses of dietary soy or soy isoflavone-rich foods and supplements. Given the heterogeneity in population and design, these studies produced interesting but divergent results. Soy products inhibited inflammatory markers in postmenopausal women [12,13,14], in women with metabolic syndrome [15], along with exercise in overweight women [16] or along with vitamin D in individuals with irritable bowel [17], in those with non-alcoholic fatty liver disease [18] and men with prostate cancer [19]. Alternatively, other studies reported no anti-inflammatory effects in healthy postmenopausal women [20,21,22], and in those with mild hypercholesterolemia [23]. Although some soy or isoflavone interventions may not have shown outcomes on inflammation markers, other cardiovascular benefits related to immune and endothelial function were noted [24,25,26].

Many traditional soy foods such as tofu, tempeh, miso, and natto are fermented products that require the involvement of microorganisms or fungi to produce. Microbial fermentation elaborates hydrolytic protease and β-glucosidase enzymes that produce bioactive peptides and convert glycosidic isoflavone glycosides into aglycones which improve the digestibility of soybeans, increasing the quantity and bioavailability of its bioactive principles, and may exert anti-inflammatory effects [27,28]. In our previous report on this clinical trial, we demonstrated that the consumption of the fermented soy powder marketed as Q-CAN^®^ Natural lowered total- and LDL-C in adult participants at cardiovascular risk [29]. In the current analysis, we explore our secondary objective which was to determine the impact of the fermented soy supplement on biomarkers of inflammation and oxidation. Also, canonical correlation analysis will be used to examine correlations between inflammatory and antioxidant biomarkers and blood lipids.

## 2. Materials and Methods

### 2.1. Study Design and Participants

A detailed description of the study design, procedures, participant recruitment, and randomization was previously described and published [29]. Briefly, the present study was conducted as a randomized placebo-controlled crossover dietary food product intervention performed in two 12-week periods with a 2-week washout between interventions. Study participants were adult men and women aged 29–75 years with at least 2 risk factors for CVD. The following were considered CVD risk factors: overweight and obesity with BMI kg/m^2^ ≥ 25; elevated systolic blood pressure ≥ 140/90 mmHg, LDL-C ≥ 110 mg/dL, triglycerides ≥ 150 mg/dL, and fasting blood glucose ≥ 110 mg/dL; low HDL-C ≤ 40 mg/dL, smoking and family history of heart disease. Exclusion criteria were any renal, hepatic, or endocrine conditions that were not being managed, allergy or sensitivity to soy or brown rice, alcohol or drug abuse, type 1 diabetes, and inability to adhere to the study protocol. Also excluded were those who were pregnant, likely to become pregnant, or lactating during the study period.

**Figure 1:**
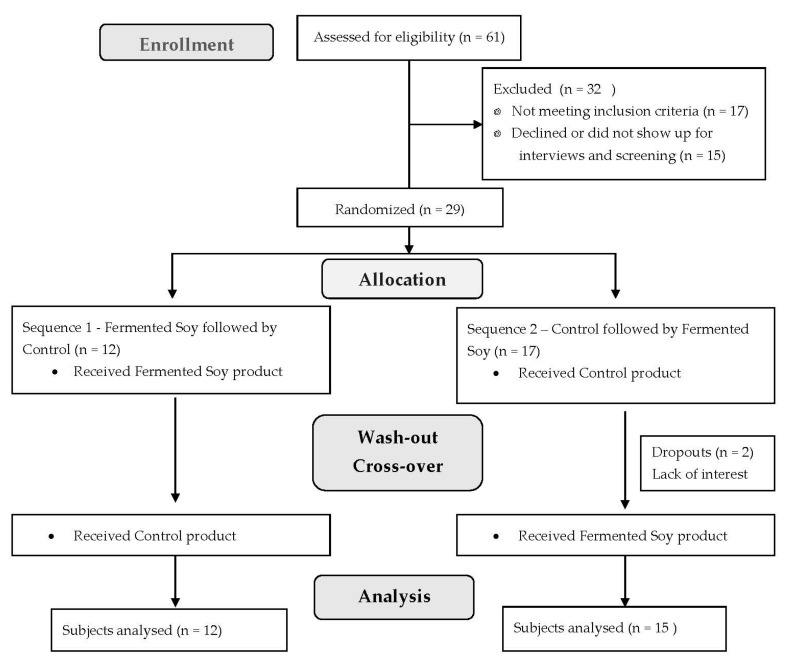
Flow chart of participants and study design. Of 61 participants who expressed interest in the study and were initially recruited, 29 were randomized and 27 completed the study.

The study included 27 participants in free-living conditions who were randomly assigned to 2 study sequences, either fermented soy powder followed by the control brown rice powder or vice versa in a typical crossover fashion. Participants were enrolled at 3 separate time points. Participants were instructed to maintain their usual physical activity levels and consistent lifestyle habits during the study period. Of 61 subjects screened, 29 were randomized and 2 participants dropped out for lack of interest. A flow chart of participants is shown in Figure 1.

The study was conducted at the Nutrition Research Center of Loma Linda University (LLU) in Loma Linda, CA. The study protocol and relevant documents were approved by the Institutional Review Board of Loma Linda University before the initiation of the trial, and participants provided written informed consent before enrolling in the study. This clinical trial was conducted according to the ethical study guidelines of the Declaration of Helsinki of 1975 as revised in 1983. The study was registered at ClinicalTrials.gov as NCT03429920.

### 2.2. Study Dietary Supplements

The intervention powder was a fermented soy product manufactured according to a patented trademarked mix of microorganisms and fermentation technology. It is marketed in the US in liquid form as Q-CAN^®^ Plus and in powder form as Q-CAN Natural^®^ by Beso Biological Research, Inc., Diamond Bar, CA, USA. The fermented soy powder contains no live cultures and was tested for toxicity and safety by an independent laboratory as reported in a recent publication [30]. The placebo powder was an isoenergic amount of dried sprouted brown rice. Both products were enhanced with chocolate flavor and monk fruit sweetener and packaged in identical packets by the research sponsor. The proximate composition of the powders has been described in our previous publication with the fermented soy powder providing approximately 36 mg of isoflavones per daily dose [29]. Subjects were instructed to consume each packet of supplement powder by mixing it with 8 ounces of either water, non-fat milk or a plant-based dairy substitute and to remain consistent with the same beverage throughout the study.

### 2.3. Laboratory Measurements

Body weight, waist circumference, body fat, and blood pressure were measured by clinicians at the Nutrition Research Center at Loma Linda University during three out of the four clinic visits for each phase of the study. Measurements were performed under a normal setting with participants wearing light loose clothing. Blood pressure was measured after at least 5 min rest using an ambulatory blood pressure monitor and appropriately sized cuffs according to the guidelines of the American Heart Association as previously explained [29]. Participants were relaxed and seated in a quiet, lighted room for 15 min before the commencement of the measurement, which lasted for 15 min and included 3 measurements. Body weight was measured within 0.1 kg using a standardized calibrated scale. Height was measured within 0.10 cm using a wall-mounted stadiometer. Body fat was measured using the TANITA bio-impedance analyzer (TBF 305) and waist circumference was taken with a non-distensible tape measure using a standard mercury sphygmomanometer.

After an overnight fast (≥10 h), blood samples were collected at baseline and the end of each 12-week period of the trial. After separation by centrifugation, serum, and plasma were aliquoted and stored immediately at −80 °C until the project was completed. Blood lipids were analyzed at the Analytical Core Laboratory, John Mayer USDA Human Research Center at Tufts University (Medford, NJ, USA) on an automated AU480 Clinical Chemistry Analyzer (Beckman Coulter, Inc., Brea, CA, USA), as specified in the manufacturer’s procedural documentation, and high sensitivity C-reactive protein (hsCRP) was measured by solid-phase, two-site chemiluminescent immunometric assays using the IMMULITE 2000, (Siemens Healthcare Diagnostics, Los Angeles, CA, USA). Tests per subject were conducted in the same analytical run to reduce systematic error and inter-assay variability. Inflammation and oxidation markers were assayed at the LLU nutrition laboratory using commercial ELISA and colorimetric kits. Tests for plasma human (IL-1), interleukin-1 receptor inhibitor (IL-1Ra), TNF-α, haptoglobin, and thiobarbituric acid reactive substances (TBARS) were assayed using ELISA kits obtained from R&D Systems (Minneapolis, MN, USA). The human IL-6 was from Abcam (Cambridge, MA, USA) and the colorimetric kit used to assay total antioxidant status (TAS) was from Millipore Sigma (Burlington, MA, USA). All samples were assayed in duplicate according to the manufacturer’s directions and absorbance was measured on an automatic microplate reader (Bio Tek Synergy HT, Winooski, VT, USA). Intra- and inter-assay CV were <10% on all assays.

### 2.4. Statistical Analysis

As previously reported [29], the study sample size was calculated considering the primary objective of the intervention which was changes in LDL-C. Between-group differences in participant characteristics at baseline were assessed by two-sample *t*-tests or Fisher’s exact tests and reported as means and standard deviations (SD) or assessed by Mann–Whitney tests and reported as medians and interquartile ranges (IQR). All inflammatory and oxidation variables were log-transformed prior to analysis to normalize their distribution and back-transformed to the original scale as geometric means. For each of the outcomes, a mixed model was fitted to compare changes from baseline to end of treatment and between treatments. The mixed models included treatment (fermented soy, brown rice), time (baseline, end), the interaction between treatment and time, sequence (1, 2), period (first, second), and enrollment set (1, 2, 3) as fixed effects terms and subjects as a random-effects term. A difference in log means was also back-transformed and expressed as a mean ratio between the two treatments.

To examine if the effect of treatment was different between sexes (male, female), a 3-way interaction of treatment × time × sex was applied. Among all markers, the 3-way interaction was significant for TNF-α and TAS which were included in the stratified analysis. Similarly, to examine if the effect of treatment was different between obese (BMI ≥ 30) and non-obese (BMI < 30) at baseline, a dichotomous BMI variable was created and added into the original model for a 3-way interaction of treatment x time x BMI. The 3-way interaction was significant for haptoglobin which was included in the BMI-stratified analysis.

Canonical correlation analysis was used to explore the inter-relationship between blood lipids and inflammatory and oxidation markers. Partial correlation coefficients were first computed between the lipid markers, the inflammatory markers, and between the lipid and inflammatory markers and adjusted for age and sex to exclude associations that were highly correlated. Canonical variates 1 and 2 were then created for lipids and the inflammatory markers and the 1st and 2nd canonical correlations between the two were computed.

Unless stated otherwise, significance tests were 2-sided and were performed at a 5% level of significance. All statistical analyses were conducted with the use of SAS software (version 9.4: SAS Institute Inc., Cary, NC, USA) and R version 3.6.3.

## 3. Results

### 3.1. Sample Characteristics

Of the 29 randomized participants, 21 females and 6 males completed the study and 2 dropped out due to lack of interest. The baseline characteristics were similar between groups as shown in Table 1.

### 3.2. Effect of Fermented Soy Supplementation on Inflammation and Oxidation Markers

Values for inflammatory and oxidation biomarkers at baseline and end of each intervention are shown in Table 2. After 12 weeks, there was a significant increase in within-treatment group levels of IL-1Ra, IL-6 and TAS in those on the fermented soy supplement and no change in any of the markers in those on the control brown rice supplement. However, between-group analysis only showed a significant effect for IL-1Ra, whereas IL-6 showed a tendency towards significance. There were no significant between-group differences in hsCRP, IL-1, TNF-α, haptoglobin, TAS or TBARS in comparing the active and control treatments.

### 3.3. Stratification of Inflammatory and Oxidative Markers by Sex and Baseline BMI

To examine if the effect of treatment differs between the sexes, we further added sex (male, female) and modeled a 3-way interaction of treatment × time × sex. Among all the markers, the 3-way interaction was found to be significant only for TNF-α (*p* = 0.018) and TAS (*p* = 0.032). Table 3 shows the outcomes of the sex-stratified analysis. TNF-α levels in female participants were lower and TAS levels in male participants were higher on the fermented soy intervention. Similarly, to examine if the effect of treatment is different between obese (BMI ≥ 30) and non-obese (BMI < 30) at baseline, we modeled a 3-way interaction of treatment x time x BMI. The interaction was found to be significant only for haptoglobin (*p* = 0.004). Haptoglobin levels were higher in those with BMI < 30 and lower in those with BMI ≥ 30 while on the control or brown rice intervention.

### 3.4. Canonical Correlation Analysis between Blood Lipids and Inflammation

To determine whether inflammatory, antioxidant, and blood lipid markers were associated in participants with CVD during the clinical trial, canonical correlation analysis was used. Canonical correlation analysis is a multivariate analysis of associations between two sets of variables, where each set consists of multiple related outcomes. It determines linear combinations of variables (called canonical variates) from each set that maximize the correlations among all possible linear combinations.

In the current study, inflammation was represented by a set of inflammatory and oxidation markers composed of hsCRP, IL-1, IL1Ra, IL-6, TNF-α, haptoglobin, TAS, and TBARS, all of which were log-transformed prior to analysis. Blood lipids were represented by LDL-C, HDL-C, and triglycerides. The loading coefficient of a canonical variate represents the contribution of each variable to the canonical variate. Table 4 shows the partial correlation matrix that includes blood lipids and inflammation markers after adjusting for age and sex. The partial correlation matrix is used to explore the inter-relationship between blood lipids and inflammatory markers.

The 1st and 2nd canonical variates between blood lipids and the inflammation–oxidation markers are shown in Table 5. The loading coefficient of a canonical variate represents the contribution of each variable to the canonical variate.

**Figure 2:**
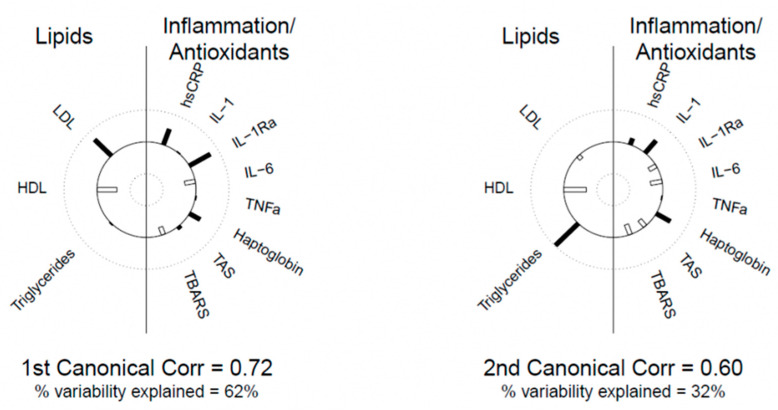
Helioplots of correlations between canonical variates and their components.

The 1st canonical variate for lipids was highly correlated with LDL-C (r = 0.715); thus, this variate was most represented by LDL-C. The 2nd canonical variate is most represented by triglycerides (r = 0.971) and both variates were negatively correlated with HDL (r = −0.605 and −0.674, respectively). For inflammation, the 1st canonical variate was represented by hsCRP and IL-1Ra, and the 2nd variate is represented by IL-1, haptoglobin, and, to a lesser degree, IL-6, and TBARS. Helioplots showing correlations between canonical variates and their components are shown in Figure 2.

The 1st correlation coefficient between the two sets of variables was r = 0.72 and the corresponding pair of canonical variates explained 62% of the variability between the biomarkers. The 2nd canonical correlation coefficient was r = 0.60 and the corresponding pair of canonical variates explained 32% of the variability. This implies that hsCRP and IL1-Ra appear to be positively associated with LDL-C and negatively with HDL-C and IL-6 and haptoglobin appears to be positively correlated with triglycerides and negatively with HDL-C.

## 4. Discussion

The current study examined the effect of a daily supplement of a commercial fermented soy powder Q-CAN^®^ on markers of inflammation and oxidation in adults with CVD risk compared to a control supplement comprised of powdered sprouted brown rice. Consumption of the fermented soy powder resulted in increased levels of the anti-inflammatory marker IL-1Ra in between-treatment comparison while increased levels of IL-6 and TAS were observed with fermented soy in within-treatment evaluation. Canonical correlation analysis showed that hsCRP and IL-1Ra concentrations were positively associated with LDL-C and negatively associated with HDL-C, and this explained 62% of the variability in the biomarkers.

Since low-grade chronic inflammation is often a feature of cardiometabolic disease, much research has focused on the anti-inflammatory effect of soy foods and their bioactive components on various cytokines and oxidation markers [31,32]. Whereas IL-1β is a key mediator of inflammation having a crucial role in the development of pathological conditions leading to chronic conditions and tissue destruction, IL-1Ra is its receptor inhibitor [33]. This randomized crossover study shows increased IL-1Ra concentration in participants with a wide range of BMIs in contrast to findings of a study using the Q-CAN^®^ beverage with no control group which reported lower IL-1Ra but only in obese subjects [30]. IL-1Ra plays an important role in the regulation of inflammatory responses [34] since mice lacking the IL-Ra gene have an increased predisposition to the spontaneous development of inflammatory disorders [35]. Additionally, IL-1Ra levels are highly associated with human obesity [33] and elevations may predict the development of metabolic disorders and type 2 diabetes [34]. In a randomized dietary intervention, IL-1Ra levels were positively associated with the intake of saturated fat and weight gain and negatively associated with the intake of magnesium [36]. Considering that participants in the current study did not gain weight, it may be postulated that the effect of the fermented soy supplement in increasing IL-1Ra levels may have produced a favorable compensatory response to low-grade inflammation in this group of participants with CVD risk.

In the current study, no fermented soy effects were found with respect to the pro-inflammatory cytokines hsCRP, IL-1β, or TNF-α; however, an increase in within-treatment IL-6 levels was noted with the fermented soy intervention and the increase approached statistical significance in between-treatment comparisons. Findings from previous randomized controlled trials with soy foods and isoflavone supplements are inconsistent reporting increased [20,37], decreased [13,14], or no change [20,21] in concentrations of IL-6. Although soy food consumption was shown to inhibit IL-6 and TNF-α in a population study [10], results from recent meta-analyses and reviews of clinical trials examining the effect of soy intake on inflammation are inconclusive and show modest or no beneficial effects of soy intake in mitigating one or more of the cytokines or inflammatory indicators [38,39,40]. Although it is unclear why IL-6 levels increased in participants consuming the fermented soy powder, the causes may be due either to participant characteristics or the soy supplement dose. In a recent subgroup analysis of trials with isoflavone containing soy products among women, serum IL-6 concentration increases occurred in studies where the isoflavone doses were ≤87 mg per day, in studies where participants had chronic disease risk factors and BMIs > 27 kg/m^2^, and in crossover studies [41]. The fermented soy supplement in the current study contained approximately 36 mg of isoflavones per daily dose and participants were obese with a mean BMI of 32 kg/m^2^. On the other hand, the fermented soy group showed an increase in TAS, a marker for the concentration of antioxidants in the blood. Soy foods are known to have antioxidant effects [42] which may have an important role in the recognized benefits of soy foods and their bioactive flavonoid components [6].

Since participants in the current study included a higher proportion of females and obese individuals, stratification analysis was applied to determine whether the results of the trial were confounded by sex or BMI. With regards to sex, TNF-α levels decreased with the fermented soy supplement only in females, and although both males and females on fermented soy showed increased TAS, a relatively greater increase occurred in males. Gender differences due to soy foods are likely due to their content of isoflavones. It is well-known that isoflavones as estrogens mimetics may exert pseudo-hormonal activity by binding to estrogen receptors in females [43]. Although some clinical trials of soy foods among women have shown reductions in TNF-α levels [12,17], findings from recent meta-analysis studies have been inconsistent [38,40]. One subgroup analysis of clinical trials reported that inhibition of TNF-α generally occurred in studies utilizing lower doses of isoflavones [31].

Stratification as a function of BMI showed an association with haptoglobin levels. Haptoglobin is an acute phase α-glycoprotein secreted mainly by the liver whose major biologic function is to strongly bind free hemoglobin, thus preventing renal and vascular injury, loss of iron, and heme-initiated oxidation of proteins and membrane lipids [44,45]. In the absence of clearance, free hemoglobin can catalyze the formation of free radicals that promote the oxidation of LDL-C [46]. In the present study, mean haptoglobin levels were higher at baseline as a function of BMI, but only the control supplement intervention showed treatment effects. The initial differences were also altered as a function of BMI following the sprouted brown rice intervention with significant haptoglobin increases in those with BMI < 30 and decreases in those with BMI ≥ 30. Overweight and obese individuals are known to have elevated plasma haptoglobin and levels are significantly reduced with weight loss [47]. Because of its role in the removal of oxidative species from the circulation [48], both high and low levels of haptoglobin are indicative of a variety of disorders with low levels associated with autoimmunity and increased levels occurring in inflammation, diabetes, and cardiovascular disease [45]. However, few studies have examined the effect of diet on this novel biomarker of systemic inflammation. Natural cocoa consumption resulted in a significant decrease in haptoglobin in obese participants [49], whereas the Mediterranean diet had no effect in a study of breast cancer survivors [50]. Our findings suggest that the sprouted brown rice powder may not be an entirely neutral control product and may contain bioactive compounds likely active in modulating biomarkers of inflammation and oxidative stress [51].

As indicated above, we previously reported on the primary outcomes of the trial and the effect of the fermented soy product in lowering total- and LDL-C [29]. Although LDL-C remains an established causal risk factor for atherosclerosis, low-grade systemic inflammation, and oxidative stress are risk enhancers and have crucial impacts on the development and progression of coronary artery disease from endothelial dysfunction to clinical syndromes [52,53], with hsCRP emerging as an independent predictor of CVD [54]. In the current analysis of the data, the association between blood lipids and inflammatory markers in our otherwise healthy participant with risk factors for CVD was examined. Since both blood lipids and the inflammatory markers are multidimensional and intercorrelated with each other, it is challenging to directly evaluate the relationship between them. Therefore, canonical correlational analysis, a multidimensional technique that can linearly combine multiple factors into groups and analyze the correlation between the two groups’ variables, was applied [55]. For the inflammation biomarkers, the 1st canonical variate was represented by hsCRP, IL-1Ra, and haptoglobin and these were positively correlated with LDL-C (r = 0.715), whereas the 2nd canonical variate was represented by hsCRP, IL-1β, and haptoglobin and correlated with triglycerides (r = 0.971). Both canonical variates were negatively correlated with HDL (r = −0.605 and −0.674, respectively). Although few nutrition and health studies apply canonical correlation to their data [56,57], these findings support evidence of a close association between blood lipids and inflammation. Chronic inflammation characterized by hs-CRP > 2 mg/L is linked to the development of CVD [58], and a large secondary prevention trial of patients who have had a myocardial infarction established that reducing inflammation with anti-cytokine therapy reduces the incidence of CVD without changes in lipids [59]. To be effective, dietary interventions must concurrently provide foods which modulate inflammation in addition to reducing atherogenic lipids.

In Asian countries, commercially fermented soy products such as Q-CAN and traditional fermented soybean foods, such as natto, tempeh, doenjang, and miso are commonly consumed and are postulated to be more effective than unfermented soy foods in mitigating lipids [60] and other atherogenic risks. Fermentation requires the involvement of microorganisms and produces a different kind of soy product often with improved nutritional value and enhanced chemical and sensory qualities [61]. The fermentation process may also inhibit some anti-nutrients found in soy and increase the digestibility of soy foods and the bioavailability of their bioactive components [62]. Soybeans are one of the richest sources of isoflavones and although native forms of soybean isoflavones are conjugated with various sugars which reduces their bioavailability, fermentation has been reported to increase the aglycone isoflavone content and increase isoflavone bioavailability [63,64]. Protein which constitutes approximately 40% of the soybean seed content may be degraded into peptides by microbial proteases during fermentation [65]. Peptides produced from soy fermentation are bioactive and exhibit various favorable effects, including antioxidant and anti-inflammatory activity [32].

Although the functional properties associated with inflammation and oxidative stress of fermented soy foods have been tested in numerous cell culture and animal studies [27,61,65], population studies and human intervention trials with fermented products are limited. Recently a cross-sectional study in Japanese workers reported an inverse association between the consumption of fermented miso and soy sauce and plasma concentration of the IL-6 cytokine in men, while dietary intake of non-fermented soy foods exhibited no such correlation [66]. In another cross-sectional study in Japanese men, higher frequencies of fermented soy product intake were associated with decreased arterial stiffness and remained so even after adjusting for serum hs-CRP, a known biomarker for systemic inflammation [67]. In a randomized placebo-controlled clinical trial, a fermented soymilk product containing *Lactobacillus plantarum* resulted in improved oxidative stress biomarkers in type 2 diabetic subjects [68]. A clinical trial with no control arm revealed that the fermented soy beverage Q-CAN^®^ Plus, similar to the one used in the current study, reduced serum levels of platelet-derived growth factor in lean subjects and IL-1Ra and granulocyte-macrophage colony-stimulating factor in obese subjects [30].

The exact mechanisms by which fermented soy foods exert their actions are unknown and underlying mechanisms can vary due to the diversity of fermented foods and products available and which are formulated with an array of microorganisms producing a multiplicity of bioactive constituents. Fermented soy benefits have been attributed to bioactive peptides and amino acids, isoflavones both glycosylated and as aglycones, saponins, anthocyanins, and to atypical bacterial metabolites and breakdown products of sugars or fatty acids as summarized in recent reviews [27,28,61,62,69]. Because the free flavonoid content of fermented products is relatively higher and shows greater bioavailability than in soybeans [70], many studies focused on the antioxidant and anti-inflammatory effects of soy flavonoids, particularly the isoflavones [69]. On a molecular level, extracts prepared from fermented soy foods and isoflavones suppress activation of the transcription factor nuclear factor- κB (NF-κB), a major effector in inflammatory and immune responses [71,72,73]. Fermented soy products have also been found to influence the Janus kinases/signal transducer and activator of transcriptions (JAKs/STATs) and the mitogen-activated protein kinases (MAPKs) pathways and to inhibit the expression of inflammatory cytokines [27,61,69]. Furthermore, constituents found in fermented foods such as bioactive peptides and saponins are also anti-inflammatory and found to moderate NF-κB [32,74,75]. Mechanisms that might explain the cardiometabolic benefits of consuming fermented soy foods may be related to the observed changes in the oral and intestinal microbiome [76,77]. Hence, even though it may not be possible to precisely identify the bioactive constituent in a fermented soy food or its mechanism of action, the outcomes observed represent the synergistic effect of all components of the product.

The strength of this study derives from the rigorous crossover design that allowed participants to act as their own control and, thereby, minimize the influence of between-subject variability when analyzing treatment effects. The study focused on participants with at least two known CVD risk factors, and most were overweight or obese but had inflammatory marker concentrations within normal ranges. However, the study had limitations. The number of subjects was determined based on the primary objective which was the effect of the supplement on blood lipids and may have been underpowered for assessing differences in inflammation and antioxidant outcomes. Several of our findings were exploratory which limits their validity and *p*-values for the analyses were not corrected for multiple comparisons which increases the risk of type I error. Despite an open recruitment policy, the participants were predominantly elderly and female which limits the generalizability of the study. The odor and taste of the fermented soy supplement were distinct; therefore, participant compliance and blinding may have been compromised. Participants were advised to mix the study powders with water or milk, but some reported difficulties in fully dissolving the powder and it is therefore possible that the full amount was not consumed. More seriously, the germinated brown rice powder chosen as the control supplement may not have been an entirely neutral product and may have had some important anti-inflammatory and antioxidant properties [51,78].

In conclusion, the current crossover intervention trial showed a 12% increase in the anti-inflammatory factor IL-1 Ra with the fermented soy product Q-CAN^®^ compared to the control powder whereas within-treatment effects of fermented soy showed 23% and 7% increases in IL-6 and TAS, respectively. The first canonical correlation between inflammation markers and blood lipids indicated hsCRP and IL-1Ra were positively associated with LDL-C and negatively associated with HDL-C, which implies a close relationship between blood lipids and inflammation. These findings indicate that fermented soy products may impact blood lipids, antioxidants, and inflammatory factors and help inform future studies on soy and inflammation.

## Figures and Tables

**Table 1:** Participant characteristics at baseline for the 27 adults with at least 2 risk factors for CVD according to randomized group assignment.

	Sequence 1 Fermented Soy → Control	Sequence 2 Control → Fermented Soy	*p* Value
	Mean (SD)	Mean (SD)	
Age, y	50.3 (12.3)	52.5 (14.8)	0.68 ^1^
Sex			
Female, n (%)	8 (66.7)	13 (86.7)	
Male, n (%)	4 (33.3)	2 (13.3)	0.36 ^2^
BMI, kg/m^2^	32.3 (9.0)	32.2 (5.9)	0.98 ^1^
LDL-C, mmol/L	3.03 (0.68)	3.07 (1.17	0.93 ^1^
HDL-C, mmol/L	1.36 (0.31)	1.41 (0.30)	0.71 ^1^
Triglycerides, mmol/L	1.35 (0.56)	1.51 (0.36)	0.53 ^3^
	Median (IQR)	Median (IQR)	
High-sensitivity C-reactive protein (hsCRP), mg/L	5.45 (1.10, 9.85)	3.90 (2.05, 7.15)	0.999 ^3^
Interleukin-1 beta (IL-1β), pg/mL	0.07 (0.02, 0.11)	0.08 (0.03, 0.12	0.598 ^3^
Interleukin-1-receptor agonist (IL-1Ra), pg/mL	771 (565, 832)	340 (309, 482)	0.143 ^3^
Interleukin-6 (IL-6), pg/mL	2.92 (0.56, 5.04)	0.50 (0.20, 2.44)	0.162 ^3^
Tumor necrosis factor-alpha (TNF-α), pg/mL	1.33 (0.63, 1.77)	1.15 (0.55, 1.99)	0.815 ^3^
Haptoglobin, pg/mL	88.1 (57.1, 110.5)	108.1 (88.1, 129.5)	0.356 ^3^
Total antioxidant status (TAS), mmol Trolox eq/L	1.75 (1.61, 1.92)	1.83 (1.68, 2.05)	0.500 ^3^
Thiobarbituric reactive substances (TBARS), μM	0.58 (0.54, 0.64)	0.55 (0.43, 0.62)	0.298 ^3^

^1^ Two-sample *t*-test; ^2^ Fisher’s exact test; ^3^ Mann–Whitney test.

**Table 2:** Serum inflammation and oxidation marker concentrations and treatment effects at baseline and the end of each of the 12-week intervention periods (n = 27).

	Fermented Soy Supplement				Control Supplement				
Variables ^3^	Baseline	End of Study	Ratio (End/Baseline)	Within *p* Value ^1^	Baseline	End of Study	Ratio (End/Baseline)	Within *p* Value ^1^	Between *p* Value ^2^
	Mean (95% CI)				Mean (95% CI)				
hsCRP, mg/L	4.07 (2.11, 7.86)	5.02 (2.62, 9.62)	1.23 (0.98, 1.54)	0.068	3.54 (1.83, 6.86)	3.76 (1.96, 7.21)	1.06 (0.85, 1.33)	0.606	0.346
IL-1β, pg/mL	0.05 (0.04, 0.08)	0.07 (0.05, 0.10)	1.30 (0.68, 2.49)	0.278	0.06 (0.04, 0.09)	0.08 (0.05, 0.12)	1.27 (0.66, 2.41)	0.336	0.929
IL-1Ra, pg/mL	475 (301, 749)	533 (338, 839)	1.12 (1.03, 1.22)	0.011	411 (261, 647)	397 (252, 625)	0.97 (0.88, 1.06)	0.450	0.022
IL-6, pg/mL	1.49 (0.68, 3.25)	3.32 (1.52, 7.28)	2.23 (1.25, 3.97)	0.007	1.86 (0.86, 4.04)	1.88 (0.87, 4.08)	1.01 (0.58, 1.78)	0.971	0.052
TNF-α, pg/mL	0.89 (0.69, 1.15)	0.77 (0.59, 1.00)	0.86 (0.64, 1.15)	0.175	0.96 (0.74, 1.24)	0.83 (0.63, 1.08)	0.86 (0.64, 1.16)	0.194	0.968
Haptoglobinpg/mL	88.6 (54.9, 143.2)	98.2 (60.8, 158.6)	1.11 (0.89, 1.37)	0.339	86.3 (53.6, 140.6)	101.4 (62.6, 164.2)	1.17 (0.93, 1.46)	0.168	0.731
TAS, mmol Trolox eq/L	1.93 (1.80, 2.08)	2.06 (1.92, 1.22)	1.07 (1.01, 1.13)	0.028	1.88 (1.75, 2.02)	1.93 (1.80, 2.08)	1.03 (0.97, 1.09)	0.348	0.370
TBARS, μM	0.60 (0.51, 0.70)	0.58 (0.50, 0.68)	0.97 (0.87, 1.08)	0.570	0.60 (0.51, 0.70)	0.66 (0.56, 0.77)	1.10 (0.99, 1.23)	0.083	0.1041

^1^ *p*-values of within-group effect of time (baseline/end of study) for each treatment. ^2^ *p* values for between-group treatment (soy/control) × time interaction. ^3^ All variables were log-transformed to normalize their distribution. Marginal means were back-transformed to geometric means and expressed as a mean ratio between the two treatments. The mixed models included treatment (fermented soy, brown rice), time (baseline, end), interaction between treatment and time (treatment × time), sequence (1, 2), period (first, second), and enrollment set (1, 2, 3) as fixed-effects terms and subjects as random-effects terms.

**Table 3:** Inflammatory and oxidative markers stratified by baseline BMI and sex.

		Fermented Soy Supplement			Control Supplement			
		Baseline	End of Study	Within *p* Value ^1^	Baseline	End of Study	Within *p* Value ^1^	Between *p* Value ^2^
		Mean (95% CI)			Mean (95% CI)			
Stratification by Sex								
TNF-α, pg/mL	Female	0.89 (0.66, 1.19)	0.73 (0.54, 0.99)	0.021	0.89 (0.66, 1.19)	0.88 (0.65, 1.20)	0.950	0.104
	Male	1.30 (0.93, 1.81)	1.32 (0.63, 2.78)	0.965	2.05 (1.47, 2.85)	1.04 (0.49, 2.17)	0.143	0.271
TAS status, mmol Trolox eq/L	Female	1.91 (1.78, 2.06)	1.98 (1.85, 2.13)	0.232	1.81 (1.69, 1.94)	1.89 (1.76,2.02)	0.185	0.911
	Male	1.91 (1.64, 2.25)	2.31 (1.97, 2.71)	0.032	2.08 (1.78, 2.44)	2.01 (1.72, 2.36)	0.658	0.063
Stratification by BMI								
Haptoglobin, pg/mL	BMI < 30	71.1 (30, 165)	73 (31, 169)	0.879	58 (25, 136)	93 (40, 218)	0.006	0.046
	BMI ≥ 30	100 (76, 130)	121 (92, 158	0.121	123 (93, 161)	99 (76, 129)	0.090	0.026

^1^ *p*-values of within-treatment of effect of time (baseline/end of study) for each treatment. ^2^ *p*-values of treatment (soy/control) × time interaction.

**Table 4:** Partial correlation matrix between blood lipids and inflammation–oxidation markers ^1^.

	LDL-C	HDL-C	Triglycerides
hs-CRP	0.292	−0.281	0.145
IL-1β	−0.148	−0.285	0.250
IL-1Ra	0.326	−0.257	−0.150
IL-6	−0.195	0.246	−0.231
TNF-α	−0.006	−0.017	−0.022
Haptoglobin	0.224	−0.311	0.316
TAS	0.154	0.114	−0.117
TBARS	−0.085	0.254	−0.193

^1^ Adjusted for age and sex.

**Table 5:** The 1st and 2nd canonical variates for lipids and inflammation.

	Canonical Variate 1	Canonical Variate 2
Blood lipids		
LDL-C	0.715	−0.133
HDL-C	−0.605	−0.674
Triglycerides	0.030	0.971
Inflammation–oxidation markers		
hs-CRP	0.518	−0.206
IL-1	0.022	0.503
IL-1Ra	0.726	−0.277
IL-6	−0.324	−0.353
TNF-α	0.029	−0.025
Haptoglobin	0.369	0.488
TAS	0.096	−0.257
TBARS	−0.251	−0.335

## Data Availability

Detailed data related to this study are available on request from the corresponding author. The data are not publicly available due to ethical restrictions.
